# Identifying undetected dementia in UK primary care patients: a retrospective case-control study comparing machine-learning and standard epidemiological approaches

**DOI:** 10.1186/s12911-019-0991-9

**Published:** 2019-12-02

**Authors:** Elizabeth Ford, Philip Rooney, Seb Oliver, Richard Hoile, Peter Hurley, Sube Banerjee, Harm van Marwijk, Jackie Cassell

**Affiliations:** 1 Department of Primary Care and Public Health, Brighton and Sussex Medical School, Watson Building, Village Way, Falmer, Brighton, BN1 9PH England; 2 Department of Physics and Astronomy, University of Sussex, Brighton, BN1 9RQ England; 3 Faculty of Health, University of Plymouth, Plymouth, PL4 8AA England

**Keywords:** Dementia, General practice, Diagnosis, Prediction, Machine learning, Early detection, Primary care, Electronic health records

## Abstract

**Background:**

Identifying dementia early in time, using real world data, is a public health challenge. As only two-thirds of people with dementia now ultimately receive a formal diagnosis in United Kingdom health systems and many receive it late in the disease process, there is ample room for improvement. The policy of the UK government and National Health Service (NHS) is to increase rates of timely dementia diagnosis. We used data from general practice (GP) patient records to create a machine-learning model to identify patients who have or who are developing dementia, but are currently undetected as having the condition by the GP.

**Methods:**

We used electronic patient records from Clinical Practice Research Datalink (CPRD). Using a case-control design, we selected patients aged >65y with a diagnosis of dementia (cases) and matched them 1:1 by sex and age to patients with no evidence of dementia (controls). We developed a list of 70 clinical entities related to the onset of dementia and recorded in the 5 years before diagnosis. After creating binary features, we trialled machine learning classifiers to discriminate between cases and controls (logistic regression, naïve Bayes, support vector machines, random forest and neural networks). We examined the most important features contributing to discrimination.

**Results:**

The final analysis included data on 93,120 patients, with a median age of 82.6 years; 64.8% were female. The naïve Bayes model performed least well. The logistic regression, support vector machine, neural network and random forest performed very similarly with an AUROC of 0.74. The top features retained in the logistic regression model were disorientation and wandering, behaviour change, schizophrenia, self-neglect, and difficulty managing.

**Conclusions:**

Our model could aid GPs or health service planners with the early detection of dementia. Future work could improve the model by exploring the longitudinal nature of patient data and modelling decline in function over time.

## Background

Dementia encompasses a range of disorders characterised by progressive decline in memory, reasoning, communication and the ability to carry out daily activities [1, 2]. The negative impact of this disorder on patients, their carers, family members and society is profound [3]. It can be hard to detect as patients may not present in healthcare clinics seeking a diagnosis. Around 850,000 people currently live with dementia in the United Kingdom (UK) [4]. Driven by population ageing this is projected to exceed 2,000,000 by 2051 [5]. With a prevalence of 7.1% in the over 65 s [5], better community care for people living with dementia is one of the great public health challenges of our era.

In the United Kingdom (UK), general practitioners (GPs) play a central role in the recognition and management of dementia in the community, and receive financial incentives for maintaining dementia registers and providing care. However, only around two-thirds of the expected numbers of patients with dementia are diagnosed [6] and recorded in GP dementia registers [7], and many of them only at an advanced stage. Data from Public Health England suggest that although diagnosis rates are increasing, they were still only 67.6% in March 2017, suggesting a third of patients are still not receiving a diagnosis [8].

Timely diagnosis for all people with dementia, who wish to have the diagnosis made, is a key objective of the UK National Dementia Strategy [2]. Timely diagnosis means that people with dementia can gain access to specialist assessment, treatment and support. Once diagnosed, patients can learn about the condition and plan for the future, which may help maximize quality of life and delay admission to care homes [9]. There is a need to improve detection and recording of dementia in UK general practice. Additional and innovative means of finding patients with dementia, based on actual local data, may improve diagnosis rates.

GPs record information about all interactions with their patients in electronic patient records (EPRs). These records consist of both structured (coded) and unstructured (free text) data entered into the patient record at the point of care. Some GP practices contribute the structured parts of their patient records in anonymised form to data warehouses such as the Clinical Practice Research Datalink (CPRD), which holds data on five million current patients [10]. Unlike traditional health research datasets, these routinely collected clinical data offer the opportunity to augment conventional health variables with multiple administrative and social variables (referrals, social care needs, etc), and with longitudinal patterns, such as changes in a patient’s symptoms or medications over time, with high external validity to the real world. These records are frequently used by researchers for epidemiological studies or for monitoring post-marketing drug safety [11].

GP patient records could provide a valuable resource for improving the detection of dementia in general practice, and may provide a practical data source for creating diagnostic support algorithms for GPs. Retrospective studies have demonstrated significant differences in signs and symptoms found in the GP records of patients leading up to a dementia diagnosis compared to patients who do not go on to develop dementia [12, 13]. Cognitive symptoms, contact with social care professionals, unpredictable consulting patterns, increased attendance, level of carer involvement, and gait disturbance were all higher in patients who went on to be diagnosed with dementia within the next 5 years [12, 13].

While many studies have attempted to create clinical risk prediction models for dementia [14–18], only a few have tried to do this using only routinely collected general practice data [19–21], and none have been focused on early detection. One example of predicting future dementia risk from primary care data was presented by Walters et al. who created a clinical prediction model for dementia using only 14 clinical variables which performed poorly (C index of 0.56) in patients over 80 years old, where risk is highest. It had good discrimination for 60–79 year olds (C index of 0.84), but various thresholds for high risk resulted in either a low sensitivity or a low positive predictive value (0.11) [19]. A German primary care cohort of people 75 years of age and over, used even fewer variables (12) in a stepwise multivariate Cox proportional hazards model, achieving an AUC of 0.79; notably this used specific assessment procedures as predictors, which may add to clinic workload in routine primary care [20].

A further limitation of the current state of evidence is that is not clear which statistical methods work best when creating models with primary care data. Machine learning approaches have been trialled for predicting dementia, using predictors such as known clinical risk factors, dementia symptoms, and behaviours (such as missing appointments) [21]. One study found that a Naïve Bayes classifier gave the best result [21]. However, it incorporated a range of clinical information indicating that the GP had already picked up on dementia symptoms (e.g. codes for forgetfulness) and gave no information about the most important features in the model. To aid early detection, and to create models which could underpin dementia diagnostic support algorithms, it is important to develop models that can detect dementia before memory loss symptoms are noted by the GP.

In contrast to previous studies, our aim was to detect existing dementia before any evidence that the GP had done so, that is, before she or he had started recording memory loss symptoms or initiating the process of dementia diagnosis. We developed models, based on routine retrospective GP data, to best predict dementia caseness detected in usual care, using information in the five years before diagnosis (or matched date in controls). We aimed to improve on previous studies by (i) incorporating previously unused symptoms, medications, social and administrative variables (“clinical entities”) as predictive features, and generating feature weights illustrating the most important predictors in the model; and (ii) comparing a range of machine learning techniques with a baseline approach of logistic regression.

## Methods

### Data source

This study used data from the UK Clinical Practice Research Datalink (CPRD) [22], established in 1987, which now contains anonymized healthcare records from more than 20 million people of whom more than five million are live in the system, representing 8% of the UK population [10]. Patients are representative of the UK general population in terms of age, sex and ethnicity. CPRD includes longitudinal observational data from GP electronic patient record systems in primary care practices, including medical diagnoses, referrals to specialists and to secondary care, primary care tests and investigations, lifestyle information (e.g. smoking, exercise) and prescribing data [10, 23]. Data are captured using a structured hierarchical vocabulary called Read codes [24]. Each Read code represents a health-related concept. There are > 200,000 different codes, which are sorted into chapters (diagnoses, processes of care and medication) and subchapters [24]. Each health-related concept is represented by a 5-byte alphanumeric code and a Read term which is the plain language description. The CPRD “Gold” dataset is drawn from the electronic patient record software Vision [25].

### Study population

Patients were selected from the CPRD database according to the following specification:

1. Patients with dementia (cases) were identified by the presence of one or more dementia diagnostic codes. We adapted code lists developed by Russell et al., [26] and Rait et al., [27] (Additional file 1). The dementia code was recorded between 2000 and 2012 and the date of the first dementia code was taken as the “index date”. Cases were 65 years or older at the index date and had up-to-standard records available for at least three years prior to diagnosis. All patients within the CPRD Gold dataset matching these criteria were extracted.
2. Control patients matched cases on age, sex, and general practice with three years up-to-standard data prior to the date of the matched case’s index date, but had no dementia code anywhere in their patient record (up to death or end of their data collection). They were randomly sampled from the CPRD Gold dataset resulting in a 1-to-1 match between cases and controls. The index date in the controls was taken from the first diagnosis code of the matched case.

Once eligible patients had been identified, the entire available coded patient record was extracted for each patient; clinical notes and letters were not available in this dataset. This resulted in records for 95,521 individuals.

**Fig. 1:**
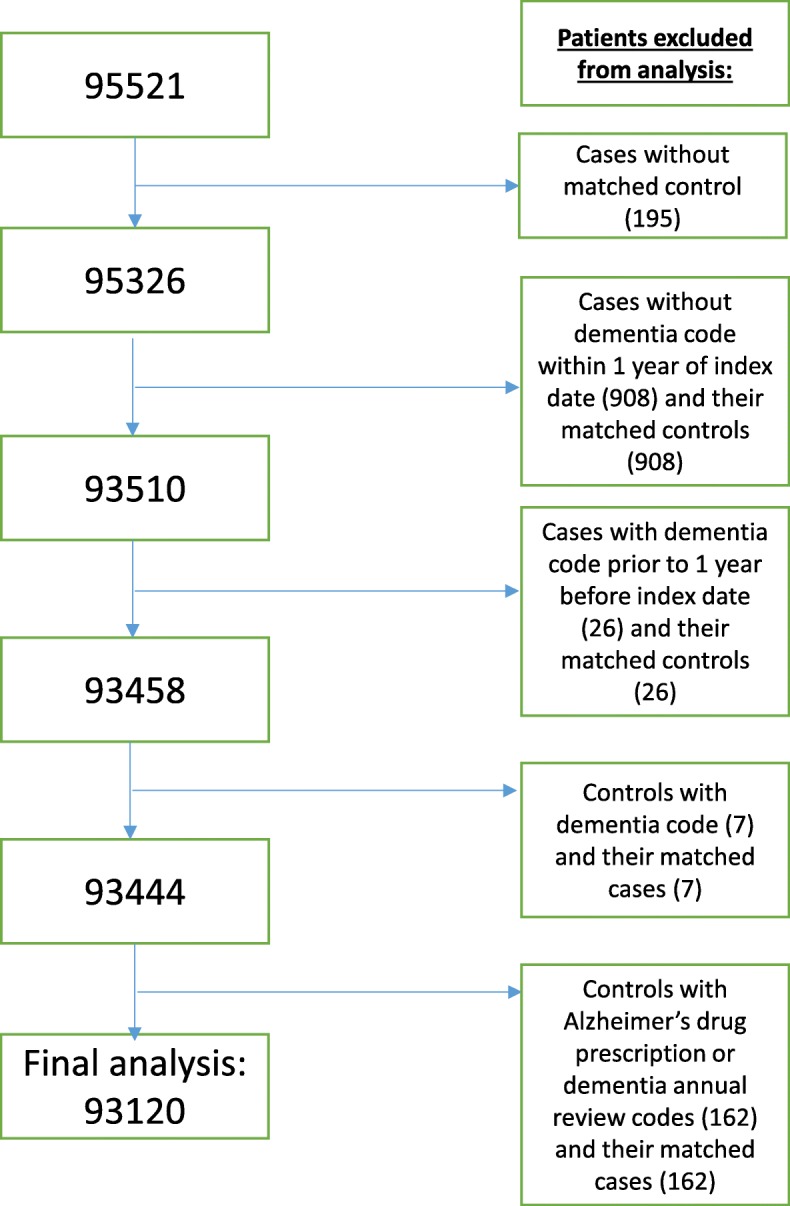
Flow chart of sample selection

The following patients were then excluded from the dataset: cases without a matched control; cases without a dementia code within one year of their assigned index date; cases with dementia codes more than 1 year prior to the index date; controls who had a dementia code; controls prescribed medication specifically for Alzheimer’s; and controls with a code for a dementia annual review. To retain the 1:1 matching, the matched case or control was also removed (See Fig. 1).

### Selection of model predictors

We defined clinical entities or features a priori for this study, because of: (i) the volume of different Read codes (60,000+ individual codes in our dataset); (ii) the fact that there may be multiple Read codes representing the same clinical entity; and (iii) the difficulty of creating meaningful clusters of codes using data-driven methods. We drew on two sources for deciding on clinical features. First we completed a systematic review and meta-analysis of potential features from primary care records research on dementia [28]. Secondly, we carried out a consultation with 21 local GPs with the following written question: “*Please could you list anything you can think of which may frequently be entered in the patient record up to 3 years before a dementia diagnosis (it does not have to be causal, just occur earlier in time than the diagnosis).”* The most commonly-reported of these were: depression/low mood (suggested by 8 GPs); problem with memory (7 GPs); Fall (6 GPs); cerebrovascular accident/transient ischaemic attack (6 GPs); a ‘Did not attend’ code (6 GPs); high blood pressure (5 GPs); forgetful (5 GPs); and anxiety (4 GPs).

Features found to be associated with dementia in the meta-analysis were mapped together with the results of the GP survey and any features which were not readily represented by a code list were discarded (such long gaps between appointments). Also discarded were features which indicated that the process of dementia diagnosis had already been initiated by the GP (such as memory loss symptoms, cognitive screening tests, or referral to memory assessment services). Read code lists were then created to define all features. We sought code lists for these features from a clinical code list repository [29] and by emailing authors of studies included in the meta-analysis. Where code lists for features were not available, new lists were drawn up using the CPRD medical and product code dictionaries application by authors EF and RH and checked by PR. This resulted in 70 code lists. (Additional file 2). Binary features were created from the code lists. The creation of binary rather than count features is thought to reduce the effect of frequency of GP visits in the data [21].

### Data split by time

Code lists were matched to event-level patient data. Only data from the period five years before the index date were used. All data more than 5 years before, or at any time after, the index date were discarded. The 5-year run up period was then split into two sections representing the last year before diagnosis (year 1), to understand proximal risk factors, and the 2–5-year period before diagnosis (years 2–5), to understand static or long-standing risk factors. We ran models with each feature’s data from year 1 and years 2–5 treated as a separate feature within the models (no shared variance was assumed).

### Data analysis

Using a set seed to ensure the same split of patients for each model, the data were split at random into 80% for training and 20% for testing. We first ran a logistic regression model with LASSO penalisation [30]. This was our baseline statistical model, as logistic regression is the usual method for binary classification in epidemiological research, and the LASSO helped us to prioritise and constrain variables added to the model and allowed us to examine feature weights. We then compared further machine-learning models against this baseline method using the following algorithms:

- Random Forrest
- Naïve Bayes Classifier
- Support Vector Machines (SVM)
- Neural Networks (NN)

Data were analysed in R version 3.4.4 using the packages GLMnet, e1071, randomforest, pROC, ROCR, ggplot, and the neural network was run in python 2.7.12 with tensorflow 1.10.1 (Additional file 3). While tuning of various model parameters was examined, as well as more complex algorithm architectures, these offered no improvements over simpler models, therefore the most simple versions of models are presented.

Each model was assessed for its ability to classify dementia cases versus controls using the Area Under the Receiver Operating Characteristic Curve (AUROC) [31]. The values of sensitivity (recall) against specificity were examined for two values: a balanced cut-off point (sensitivity and specificity weighted equally) and a fixed specificity of 0.95, chosen because in the clinic, it may be important to minimise false positives. Because of the case-control design of this study, we had an artificial prevalence of dementia of 50% in our sample. We thus calculated positive predictive value (precision) of each model based on the UK prevalence of dementia of 7.1% in people over 65 years [5].

The features retained within the logistic regression models following LASSO penalisation were examined, to identify the key features of the model. These were identified by generating each feature’s logistic regression parameter, identified as β in the following logistic regression equation, where X_n_ indicates each feature:

$$ Ln\left(\frac{P}{1-P}\right)={\beta}_0+{\beta}_1{X}_1+{\beta}_2{X}_2+...+{\beta}_k{X}_k $$

## Results

### Study population

Our final sample consisted of 93,120 patients of whom 32,800 (35.2%) were men and 60,320 (64.8%) were women; 50% of the sample had one or more codes for dementia. The median age at index date was 82.6 years (range: 64.5–109.9 years). The median amount of time before index date available in the records was 19.3 years (range 3.00–102.2 years of registration). Dementia cases had a median of 161 events recorded in their whole record (range 2–2709) and controls had a median of 157 events recorded (range 0–2710). All patients had at least three years’ worth of data (100%), 90,351 patients (97.0%) had at least four years and 87,876 patients (94.4%) at least five. 70 clinical variables were included in the model as predictors.

### Logistic regression and machine learning model performance

**Fig. 2:**
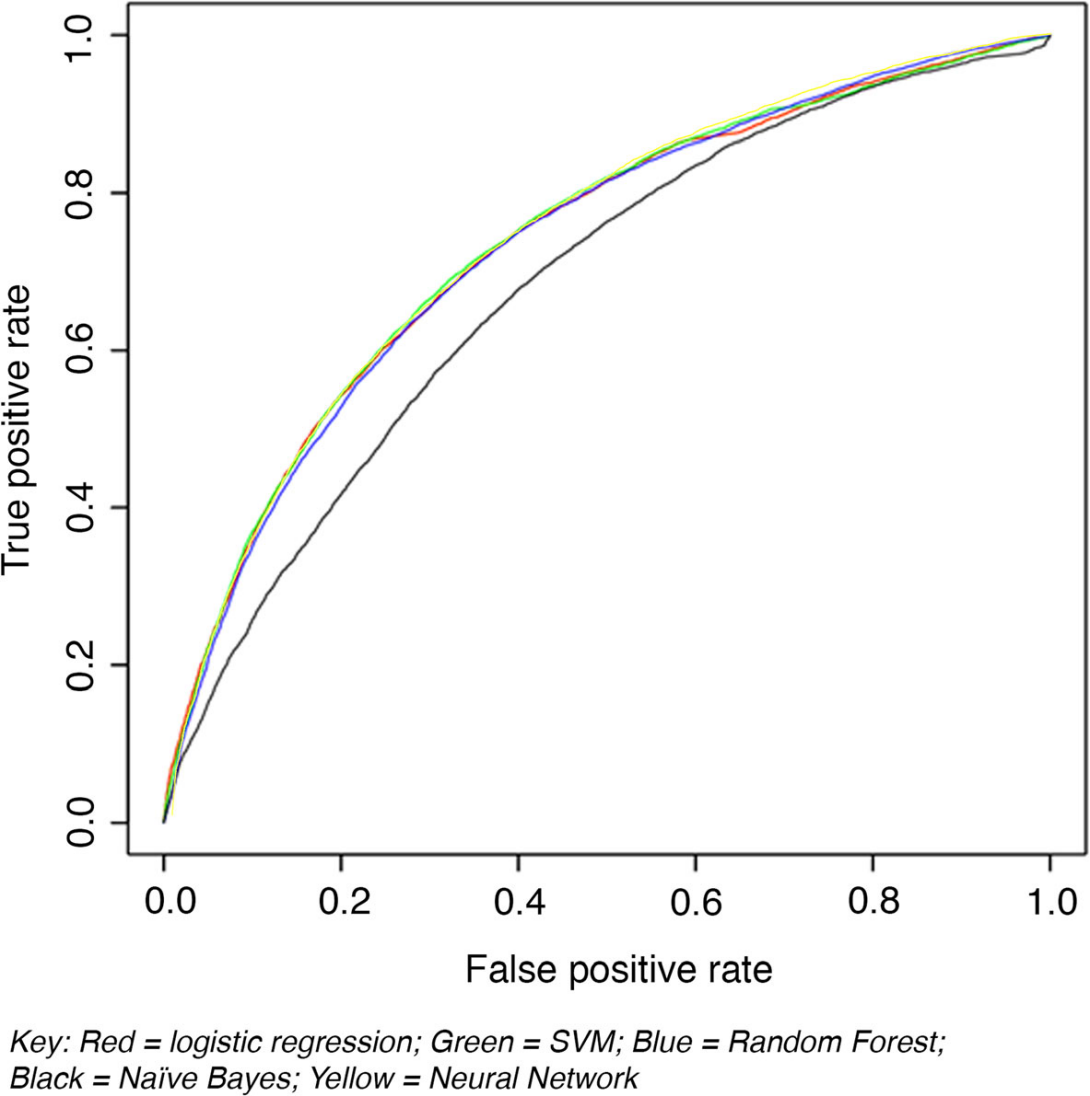
AUROC for all ML models superimposed; 1, 2–5 year data

As shown in Table 1, the logistic regression model and four further types of machine-learning models were run. Results of models can be seen in Table 1 and Fig. 2. The best AUROC was 0.74, which was achieved by the logistic regression, the neural network and support vector machine, with the random forest model performing very similarly. The Naïve Bayes classifier model was less accurate (AUROC 0.68). The neural network gave the best specificity for a reasonable sensitivity, and thus the highest PPV.

**Table 1:** Model performance (AUROC, best sensitivity and specificity, PPV)

Model Type	Time split	AUROC (95%CI)	Specificity (balanced model)	Sensitivity (balanced model)	PPV (balanced model)	Sensitivity for 95% specificity	PPV at 95% specificity
Logistic Regression with Lasso	1, 2–5	0.736 (0.728–0.743)	0.752	0.602	0.156	0.222	0.254
Naïve Bayes Classifier	1, 2–5	0.682 (0.675–0.690)	0.906	0.241	0.164	0.153	0.189
Support Vector Machine	1, 2–5	0.737 (0.730–0.744)	0.691	0.674	0.142	0.223	0.255
Random Forest	1, 2–5	0.734 (0.726–0.740)	0.653	0.700	0.134	0.210	0.239
Neural Network (3 × 139 nodes)	1, 2–5	0.737 (0.730–0.743)	0.781	0.619	0.178	0.298	0.312

### Feature weights in logistic regression model

When the features retained by the LASSO penalisation were examined, the most important features were disorientation and wandering, behaviour change, schizophrenia, self-neglect, difficulty managing, personality change and family history of dementia; the most significant features were all recorded in the final year before diagnosis. Psychotic depression and cancer were strongly negatively associated with dementia (Table 2).

**Table 2:** Features retained in Logistic Regression with Lasso Penalisation, 1 year and 2–5 years separated

Feature name	Logistic regression parameter	
1 year prior to diagnosis/matched date	1 year prior to diagnosis/matched date	2–5 year predictors
Disorientation and Wandering	2.31	0.88
Behaviour change	1.99	0.65
Schizophrenia	1.53	–
Self-neglect	1.45	–
Difficulty managing	1.38	–
Personality change	1.18	0.58
Family history of dementia	1.14	–
Third party consultation	0.85	–
Antidepressant	0.81	–
Antipsychotic medication	0.76	−0.11
Cerebrovascular disease	0.58	0.14
Did not attend	0.56	0.22
GP home visit	0.55	−0.11
Bipolar disorder	0.51	− 0.11
Interaction with social services	0.51	–
Possible Fall	0.47	0.22
Alcohol	0.42	–
Unable to cope	0.41	0.21
Attended Emergency Department	0.39	–
Depression	0.34	–
Living in a nursing home	0.31	–
Receiving care in home	0.28	–
Epilepsy or Seizures	0.23	0.25
Blood pressure measurement	0.16	–
Stroke	0.15	–
Routine hospital admission	0.15	−0.14
Z-drugs	0.13	−0.11
Lower limb fracture	0.12	–
Receiving care in home	0.11	–
Anxiety	0.10	–
Impaired mobility	0.10	–
Needs help with activities of daily living	−0.11	−0.30
Dressing of wound, burn or ulcer	−0.13	–
Family bereavement	−0.16	–
Hypertension	−0.20	−0.16
Infections	−0.21	−0.16
Angina	−0.22	–
Vertebral collapse	−0.27	–
Lithium	−0.28	–
PTSD reaction	−0.46	–
Cancer	−1.06	–
Psychotic Depression	−1.11	–
Personality disorder	–	0.21
Constipation	–	0.10
Coronary Heart Disease	–	−0.12
Obesity	–	−0.16
Benzodiazepines	–	−0.22

## Discussion

### Summary of findings

Our study gives new insights into the possibilities of identifying undetected cases of dementia in primary care by using GP patient records as the sole data source. We found that LASSO penalised logistic regression, support vector machine, neural network and random forest models performed very similarly, with a best AUROC of 0.74, although the neural network produced the highest PPV (precision; 0.31). Logistic regression and random forest algorithms may nevertheless offer an advantage over support vector machines and neural networks as they produce easy to interpret feature weights, which may be of value in a clinical situation.

### Important features in the model

In this study, the important features found by the logistic regression were intuitively important clinically and were either symptoms which indicated the patient was already in the prodromal stages of dementia or indications of increasing frailty. Symptoms such as disorientation and wandering, behaviour or personality change; medications such as antidepressants and antipsychotics; observations such as self-neglect and difficulty managing; and administrative codes such as ‘third party consultation’, ‘did not attend’ and ‘GP home visit’, were all among the top 15 features. In this regard our study is novel, compared to other primary care dementia risk prediction models, due to its expanded list of symptom and administrative features. Our aim was to identify undetected, but current cases of dementia, rather than predict onset at some future time. We thus took an approach of using clinical entities that may be associated with dementia for any reason, appearing in the patient record prior to or around the time of dementia onset, rather than restricting ourselves to entities with a causal relationship to dementia. Future work could examine at which time point, prior to dementia diagnosis, each of these features starts contributing significantly to the model.

### Performance of machine-learning over traditional methods

Our study offers an improvement on previous models which aim to predict or detect dementia using GP patient record data as the only source of information, by using an expanded list of predictors and achieving a best PPV of 0.31. Walters et al. [19], retained 14 clinical and demographic variables using Cox proportional hazards regression with backwards elimination in a cohort design. Their model showed similar sensitivity to ours but higher specificity, and a best PPV of only 0.11. Their model included age as one of the features, which is likely to be one of the best predictors of dementia, and which our matched case-control design did not allow for. Our model may thus show better performance if replicated in a cohort sample, adding in age as a predictor.

We found that machine-learning models showed no improvement over a logistic regression method which was allowed to select features using a data-driven mechanism. Some machine learning techniques allow for non-linear effects to be learned in the data, whereas logistic regression assumes linear relationships between variables. This freedom of the models to find non-linear effects did not seem to improve the discriminatory power. It may be that electronic health records data are too noisy to achieve much improvement by using newer methods over traditional methods, or it may be that all the relationships between variables in the model are best approximated with linear relationships.

With no better performance of one model over another, it is worth considering that clinicians appreciate knowing the reasons behind decisions reached by computer-aided decision support algorithms [32], to allow them autonomy and flexibility in the use of such algorithms [33]. Indeed, the new legislation on use of personal data (GDPR) may require that decisions based on processing of personal or patient data allow for transparent interpretation of how results are reached [34]. Thus the “black box” of the neural network could prove a barrier to clinical implementation [35]. While there are ways of recovering the choices or reasoning behind neural networks, these are not yet robust or reliable, especially in EHR data. Logistic regressions and random forest algorithms allow for important features to be exposed, thus aiding clinical interpretation of the algorithm classification decision, and may be the best approaches for prediction tasks which aim to be implemented in the clinic.

### Clinical implications

Our findings are a useful development of the evidence base for generating a system that can be applied to identify undiagnosed cases of dementia from primary care electronic records. Our broad approach and the elements in our model can be used and contribute to further research to create a detection tool for GPs, commissioners, or public health service planners. Our findings, taken with that generated by other groups using similar methodologies, make clear that an algorithmic approach alone is not able to make the diagnosis of dementia or identify those with dementia by itself. Future approaches are likely to use systems such as this to flag up cases where GPs can offer further clinical evaluation at the patient’s next primary care consultation. The role of primary care at this point might be to identify cases that would benefit from definitive assessment in a Memory Assessment Service, taking account of patient preference for such an assessment. After further development of our model, the next step could be pilot testing of an implemented decision support tool that is triggered to ask a GP to consider a review, request a diagnosis code, or ask the GP to ask a patient about falls and other safety issues, or even fill in more detail in the record to improve the risk estimate. A further potential use would be for service planners who wish to estimate local area prevalence of dementia, so that Memory Assessment Services can be commissioned appropriately. The next steps in developing this model should include consultation with general practitioners, patients, and commissioners, to understand stakeholder priorities for improving the model for implementation and early detection. One priority for such stakeholders might be to have the model produce a personalised risk estimate for dementia. This would mean that the response by the GP could be tailored to the patient’s individual circumstances, rather than a one-size-fits-all approach.

### Strengths and limitations

The key strength to our approach was the comprehensive strategy for identifying a wide range of potential features for the model, from clinically related diagnoses, health events and symptoms, to more social or administrative features, which may be recorded as the GP takes care of the wider needs of the patient.

Limitations include the case-control design. A cohort design would have been closer to a real life or clinic setting in terms of prevalence of dementia; in addition, age could have been included as a predictor. The model presented here should be replicated in a cohort dataset to examine its fit to a novel data set, and to refine it further. A second limitation is that static binary features may have resulted in a loss of information, although where we have previously trialled “count” features, these have not improved the accuracy of our models. Other studies have also favoured binary features in order to reduce the influence on the data of the number of times a patient visits their GP [21]. A third limitation is that we did not inform the model that variables representing the same feature measured at different times were related to each other. This could be achieved by using a multi-level model which treats yearly features as a cluster of predictors which share variance.

### Future directions

Using a comprehensive list of features we achieved a fair discrimination between cases and controls which could aid with local prevalence estimates and, particularly, estimates that are based on detailed local information, and early detection. Given our methodical approach to selecting predictive features, we believe that this model provides a strong basis for further development with more sophisticated feature engineering. Our team’s future work will explore longitudinal information within patient records to identify how much earlier the diagnosis could be made, and how the best set of features evolve in the time period before diagnosis. We will also explore the inclusion of features which indicate a change in memory or cognitive function over time, such as missed appointments becoming more common.

Many cases of dementia which are apparently undiagnosed are actually detected by GPs but unlabelled due to a lack of a formal diagnosis. These ‘detected but unlabelled’ patients may make up a substantial proportion of undiagnosed patients with dementia, as many GPs are not convinced of the benefit of a formal dementia diagnosis [36, 37]. Creating a model to find these unlabelled cases may allow for more sensitive detection of participants for clinical trials, as well as improving the quality of GP record keeping for audit, health service planning and prevalence studies.

## Conclusions

We successfully discriminated between dementia cases and controls using only features from the primary care record which did not indicate that memory problems had already been detected by GPs. We found no advantage of newer machine learning techniques over logistic regression. We identified the most important features for detecting dementia in such a model, these were found to be possible prodromal symptoms and indications of increasing frailty. With further development and as part of a comprehensive diagnostic pathway, this model may aid GPs and health service planners with the early detection of dementia in primary care.

## Supplementary information

**Additional file 1.** Dementia Read Codes.

**Additional file 2.** Feature List.

**Additional file 3.** Model Specifications.

## Data Availability

The data that support the findings of this study are available from Clinical Practice Research Datalink (CPRD; www.cprd.com) but restrictions apply to the availability of these data, which were used under license for the current study, and so are not publicly available. For re-using these data, an application must be made directly to CPRD.

## Abbreviations

AUROC: Area Under the Receiver Operating Characteristic Curve
CI: Confidence Interval
CPRD: Clinical Practice Research Datalink
EPR: Electronic Patient Record
GDPR: General Data Protection Regulation
GP: General Practitioner/General Practice
LASSO: Least Absolute Shrinkage and Selection Operator
MMSE: Mini-Mental State Examination
NHS: National Health Service
NN: Neural Network
PPV: Positive Predictive Value
SVM: Support Vector Machine
UK: United Kingdom of Great Britain and Northern Ireland
