# Supplementary material for: Identifying undetected dementia in UK primary care patients: a retrospective case-control study comparing machine-learning and standard epidemiological approaches
Source: BMC Med Inform Decis Mak. 2019 Dec 2;19:248. doi: 10.1186/s12911-019-0991-9 (PMC6889642; doi:10.1186/s12911-019-0991-9)
Supplement: Supplementary file 3 — Additional file 3. Model Specifications. [file 12911_2019_991_MOESM3_ESM.docx]

| **Model** | **Software and Package** | **Settings or Network Architecture** | **Parameters or Features** |
| --- | --- | --- | --- |
| Logistic Regression | R v3.4.4; GLMnet | LASSO set at λ = 1 | All features entered from Appendix 2 for both Year -1 and Years -2 to -5. |
| Naïve Bayes Classifier | R v3.4.4; e1071 | No parameters tuned | All features entered from Appendix 2 for both Year -1 and Years -2 to -5. |
| Support Vector Machine | R v3.4.4; e1071 | Probability = TRUE  Family = Binomial  Prob.model = TRUE | All features entered from Appendix 2 for both Year -1 and Years -2 to -5. |
| Random Forest | R v3.4.4; randomforest | Family = Binomial  Prob.model = TRUE | All features entered from Appendix 2 for both Year -1 and Years -2 to -5. |
| Neural Network | Python 2.7.12; tensorflow 1.10.1 | Architecture of 3 layers with 139 nodes in each (representing all features at 2 time points) | All features entered from Appendix 2 for both Year -1 and Years -2 to -5. |
